# Comparative prevalence of *Oxalobacter formigenes* in three human populations

**DOI:** 10.1038/s41598-018-36670-z

**Published:** 2019-01-24

**Authors:** Amanda PeBenito, Lama Nazzal, Chan Wang, Huilin Li, Melanie Jay, Oscar Noya-Alarcon, Monica Contreras, Orlana Lander, Jeff Leach, Maria Gloria Dominguez-Bello, Martin J. Blaser

**Affiliations:** 1 Department of Medicine, New York University School of Medicine, New York, NY USA; 2 Department of Population Health, New York University School of Medicine, New York, NY USA; 3 Instituto de Medicina Tropical, Universidad Central de Venezuela, Caracas, 1051 Venezuela; 4 Amazonic Center for Research and Control of Tropical Diseases (CAICET), Puerto Ayacucho, 7101 Venezuela; 5 Center of Biophysics and Biochemistry, Venezuelan Institute of Scientific Research (IVIC), Caracas, 1020A Venezuela; 6 Human Food Project, Terlingua, TX USA; 7 Department of Twin Research and Genetic Epidemiology, Kings College, London, UK; 8 Department of Biochemistry and Microbiology, Rutgers University, New Brunswick, NJ USA; 9 Department of Microbiology, New York University School of Medicine, New York, NY USA; 10 Present Address: Amanda PeBenito, Massachusetts General Hospital, Boston, MA USA

## Abstract

There has been increasing interest in the human anaerobic colonic bacterium *Oxalobacter formigenes* because of its ability to metabolize oxalate, and its potential contribution to protection from calcium oxalate kidney stones. Prior studies examining the prevalence of this organism have focused on subjects in developed countries and on adults. Now using *O*. *formigenes-*specific PCR, we have compared the prevalence of these organisms among subjects in two remote areas in which modern medical practices have hardly been present with a USA group of mothers and their infants for the first three years of life. Among the Amerindians of the Yanomami-Sanema and Yekwana ethnic groups in Venezuela and the Hadza in Tanzania, *O*. *formigenes* was detected in 60–80% of the adult subjects, higher than found in adults from USA in this and prior studies. In young children, the prevalence was much lower in USA than in either tribal village. These data extend our understanding of the epidemiology of *O*. *formigenes* carriage, and are consistent with the hypothesis that the rising incidence of kidney stones is associated with the progressive loss of *O*. *formigenes* colonization in populations that have been highly impacted by modern medical practices.

## Introduction

*Oxalobacter formigenes* are gram-negative anaerobes that inhabit the intestinal tract of humans and other mammals^1,2^. *O*. *formigenes* is unique in that it utilizes oxalate as its sole carbon and energy source^3,4^, which it metabolizes into formate and CO_2_^5^. In mammals, oxalate is an end-product of metabolism and must be eliminated either in the intestine or via urinary excretion^6,7^. The renal excretion can lead to nephrotoxicity, including calcium oxalate kidney stones^8^; thus, regulation of oxalate homeostasis has important pathophysiologic consequences.

One hypothesis is that intestinal colonization with *O*. *formigenes* helps protect against calcium oxalate nephrolithiasis^9,10^. *O*. *formigenes* utilizes dietary oxalate, reducing the amount absorbed into the circulation, and recent evidence suggests that it induces colonic oxalate transporters (SLC26), acting as a sink for systemic oxalate^11–14^. Studies comparing adults with recent nephrolithiasis and healthy adults showed a lower rate of *O*. *formigenes* colonization in the stone formers, consistent with a protective role for the organism^10,15^. Since ~75% of kidney stones are composed of calcium oxalate^16^, and since nephrolithiasis incidence is substantially increasing in both adults and children^17–19^, a focus on *O*. *formigenes* may be of interest for therapeutic applications.

Prior studies of the prevalence of *O*. *formigenes* colonization showed considerable differences, which might reflect true variation or technical issues. Studies in healthy adults suggest prevalence in the United States is between 31–38%^10,20,21^ but as high as 60–77% in Korea, Japan, and India^22–24^. Such variation could also reflect medical practice differences, as *O*. *formigenes* is known to be susceptible to many commonly prescribed antibiotics^25^. In one prospective study, antibiotic treatment resulted in lasting suppression of *O*. *formigenes*^26^. Patients with cystic fibrosis or inflammatory bowel disease, who receive multiple antibiotic courses also have lower *O*. *formigenes* prevalence than controls^27,28^. However, little is known about the transmission of *O*. *formigenes* and its timing, and the factors influencing colonization.

To further address these questions, we examined *O*. *formigenes* colonization in a small cohort of USA mothers and their infants longitudinally from birth through three years of life. To investigate the hypothesis that modern practices may be impacting *O*. *formigenes* colonization, in parallel, we examined adults and children in two remote populations: the Hadza, a tribe of hunter-gatherers in Tanzania^27^, and a group of remote Amerindians in Venezuela^29^.

## Results

### *O*. *formigenes* prevalence in the USA cohort

**Figure 1:**
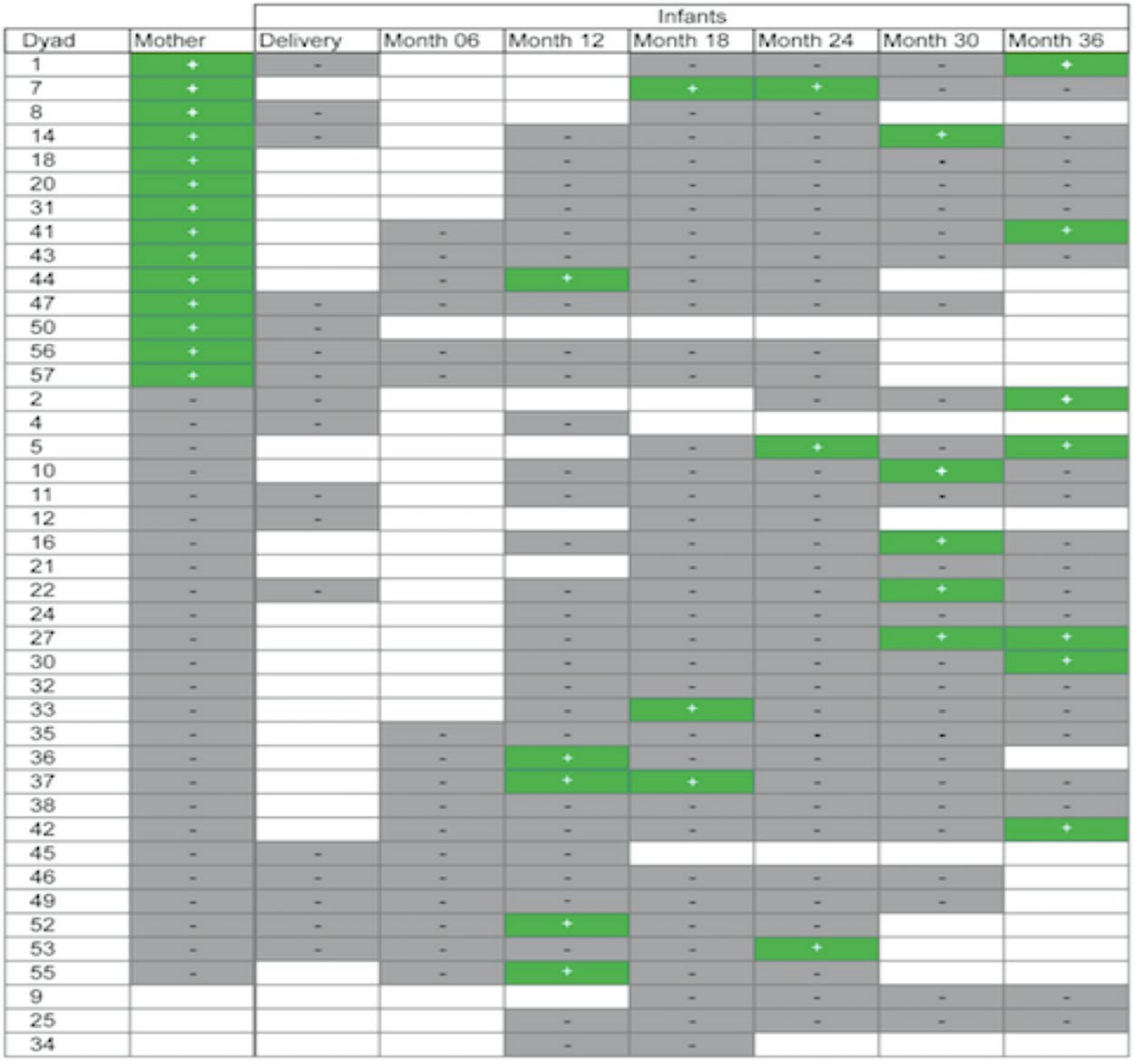
Temporal distribution of *O*. *formigenes* in mother-infant dyads from USA. Figure represents 199 samples tested from 42 individual children. In total, 19 (45%) children were positive at ≥1 time point. Four (10%) children were positive at ≥2 time points.

Among the 40 women who provided post-partum fecal samples, *O*. *formigenes* was detected in 15 (38%) (Table 1). Among the 42 infants, a total of 19 (45%) were positive at least once in the multiple determinations, and 4 (10%) were positive at least twice (Fig. 1). *O*. *formigenes* was only found in children greater than 1-year old and not in infants, and the prevalence appears to increase with age, although study numbers are small.

**Table 1:** *O*. *formigenes* in USA maternal and infant fecal samples by PCR.

Sample	N	% Positive
Mothers	40	38
Infants		
Post-Delivery	17	0
Year 1	50	10
Year 2	76	8
Year 3	56	21

**Figure 2:**
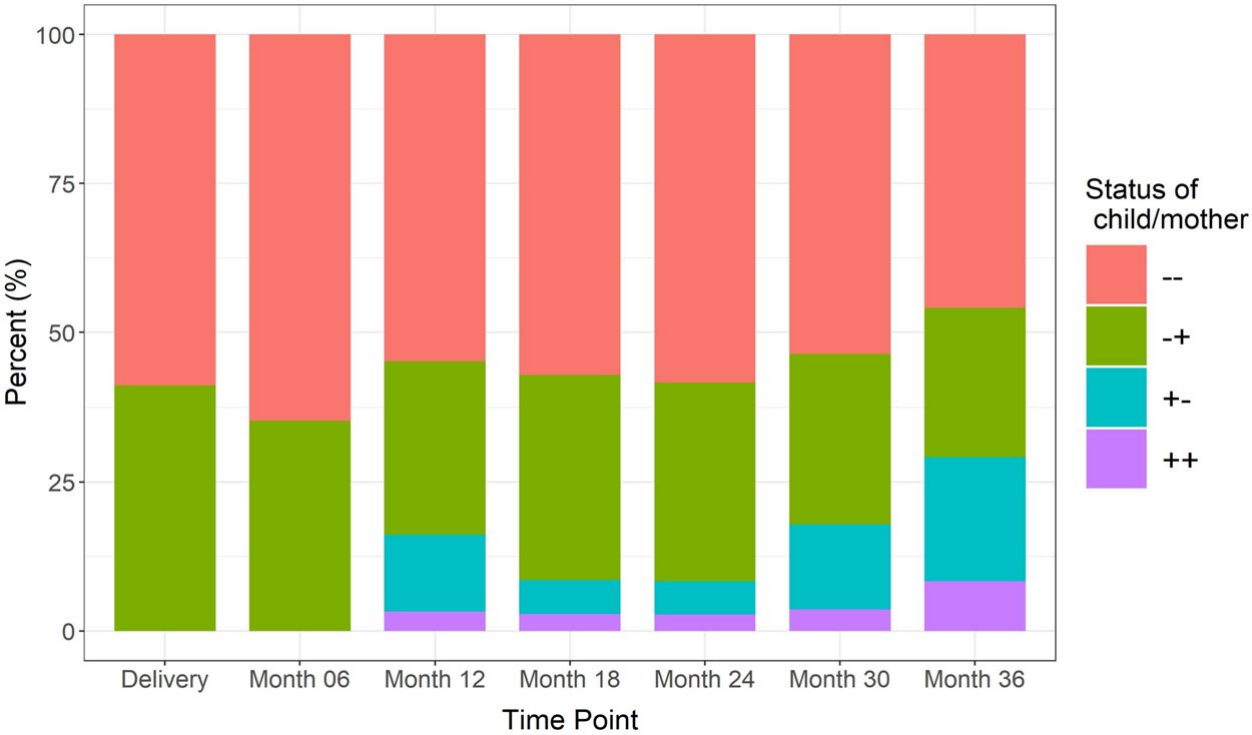
Concordance of maternal and child *O*. *formigenes* status over the first three years of life. *O*. *formigenes* detected by *oxc* PCR, as described in the Methods.

We then assessed whether there was a relationship between maternal and infant colonization status, using logistic regression with and without adjustments for maternal covariates (age, BMI, and race), calculating Cohen’s kappa statistic to examine the agreement between maternal and infant PCR results. We also used a generalized linear mixed model to analyze PCR results with or without adjustment for those covariates as well as maternal antibiotic use prior to pregnancy, during pregnancy, and during delivery. There was no statistically significant correlation between maternal and infant colonization, whether evaluating infant colonization at each time point or in infants positive at any point by either model (Fig. 2). Next, we asked whether *O*. *formigenes* status of the infants was correlated with their delivery method or gender. First, we used Fisher exact test and chi squared tests to assess the correlation between these variables and infants’ PCR results. Logistic regression and the generalized linear mixed model were also applied. We found no significant correlation between infant delivery method or gender and their *O*. *formigenes* colonization status at individual time points or positivity at any time, with or without adjustment for maternal covariates. We also asked whether *O*. *formigenes* colonization status was correlated with infant antibiotic use. We examined whether antibiotics given perinatally (intravenous antibiotics given to the newborn), systemic antibiotics given during the first, second, or third year of life, or systemic antibiotics given at any point correlated with infants’ *O*. *formigenes* status. Again there was no significant correlation between antibiotic use and colonization at subsequent time points by logistic regression or generalized mixed model with or without covariate adjustment.

### O. *formigenes* prevalence in hunter-gatherer populations

**Figure 3:**
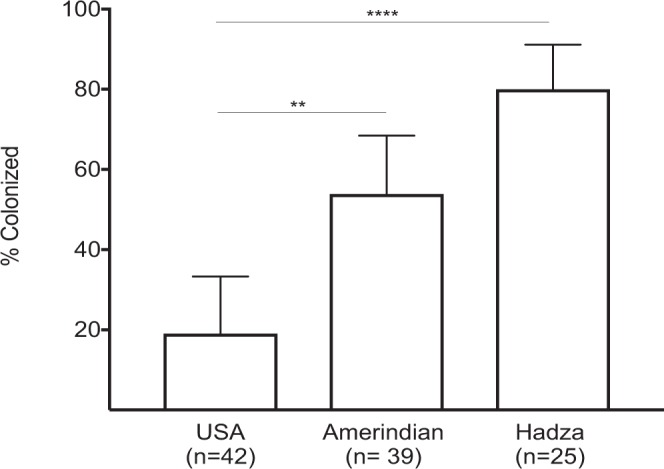
Percent of children less than 5 years old positive for *O*. *formigenes* in three populations. Only single samples were present from the Amerindian and Hadza children. Multiple samples were tested for the USA children, but only the sample from each child at the oldest age tested was used for the calculation. Percent of children colonized, with 95% CI shown (**p < 0.01; ****p < 0.001, by Fisher’s exact test).

**Figure 4:**
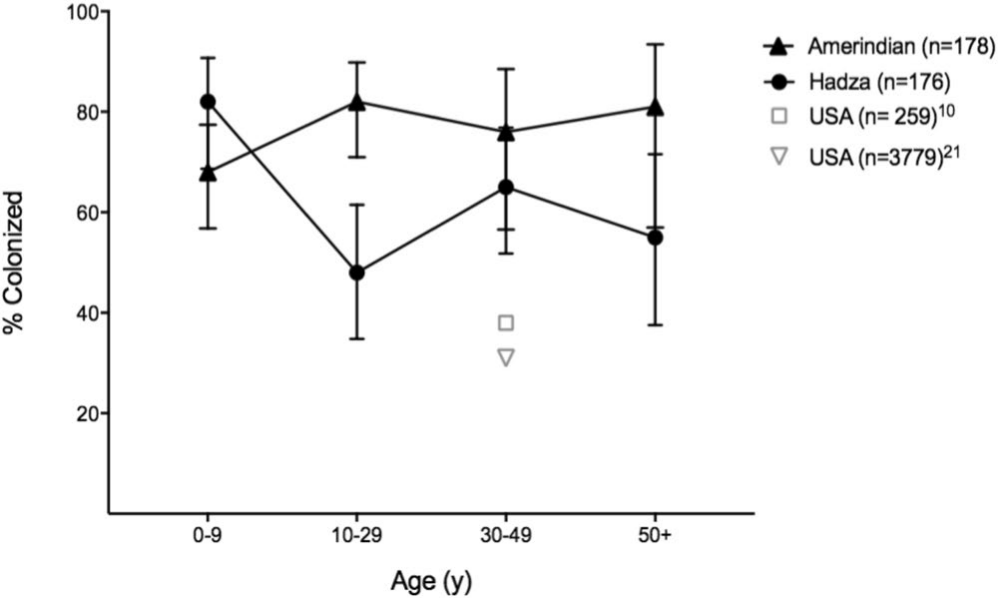
*O*. *formigenes* prevalence in traditional and USA populations, by age. Positivity for the Hadza and Amerindians determined by end point PCR using *O*. *formigenes*-specific primers, as described in Methods. Results for two prior US studies for comparison using different methodologies are shown at ~the mean age of the populations tested with the percent colonized indicated; the mean ages were 42 and 46, respectively^10,21^.

We compared prevalence of *O*. *formigenes* colonization in the cohort of US children with that in tribal populations from Africa and South America. US children had the lowest prevalence (19%) (Fig. 3); compared with Amerindians (68%; p = 0.0013), and with Hadza, (82%; p < 0.0001, Two-sided Fisher exact test). There were no significant differences in prevalence between the Amerindian and Hadza children. While there was no evidence of colonization in the USA children until 12 months of age (0/17 were positive at 6 months (Fig. 1), *O*. f*ormigenes* was detected in some of the youngest subjects sampled in the Hadza and Amerindian populations: the earliest at 9 months and 3 months of age respectively (data not shown). We also examined *O*. *formigenes* prevalence in adulthood in 68 Amerindian and 116 Hadza subjects. Among the adults, 79% of Amerindians and 55% of Hadza subjects were PCR-positive. Comparing the two populations by age group (Fig. 4) demonstrated similar rates of colonization except among adolescents and young adults, in which prevalence in the Hadza was significantly lower. Regardless, the prevalence of *O*. *formigenes* among both healthy Amerindians and Hadza was substantially higher than that of healthy adults in the United States (ranging from 31% to 38%), as reported in three large and independent studies^10,20,21^, as well as the US mothers in this work.

## Discussion

To our knowledge, this is the first study to examine early life *O*. *formigenes* colonization in more than 20 years and the only study that has examined a cohort of mothers and children longitudinally from birth through the first years of life. The other study of *O*. *formigenes* colonization in early life was a cross-sectional evaluation of children ages 0–12 in a remote area of the Ukraine where antibiotic use was considered to be limited^30^. In that study, Sidhu *et al*. found that 72% of children were PCR-positive, with no evidence of colonization before the age of one year, but reaching 90% by age 2; nearly all children appeared to be colonized by age 8, after which rates declined. Similar to the findings in Ukrainian infants, we found no evidence of colonization in children less than one year in our U.S. cohort. This may be explained by *O*. *formigenes* acquisition from the environment, as infants begin to become more mobile at this age, as proposed^30^ and demonstrated in rodent models^31^. The lack of association between maternal and infant colonization in this study also supports the hypothesis of horizontal transmission. Alternatively, *O*. *formigenes* may be acquired at birth from mothers but only at low abundance, blooming later in life with advancement of infants’ diet or with maturation of the microbiome and the acquisition of commensal species required for *O*. *formigenes* colonization success. The current study did not provide any direct support for this hypothesis, but more prolonged cohort studies with a larger number of subjects may be needed to better address this question. All of the children who were found to be *O*. *formigenes*-positive, also had negative samples at the earliest time points. Our past work^20,21^ showed that the relative abundance of *O*. *formigenes* in fecal specimens varies over >3 log_10_. Children with low abundances may be near the threshold of detection, with the variation in positivity that we observed consistent with our prior findings^21^.

Several studies have examined the intestinal microbiome of the Hadza^29,32–34^ and to a lesser extent, the Amerindians of Venezuela^35^. Since these groups have had only recent exposure to Western medicine, their results provide a window on the pre-industrial intestinal microbiome of humans. Similar to our prior study of a small uncontacted Amerindian population using high throughput sequencing, the prevalence of *O*. *formigenes* was substantially higher than that of USA subjects^35^. Now we provide evidence of higher rates in a larger population of both children and adults in remote Amerindian and African populations than in the USA, including those in this study as well as in the literature^10,21,36^. A possible explanation for this difference is that developments associated with the Westernization, such as changes in diet or antibiotic use may contribute to the loss of this commensal organism. A recent report of the association of kidney stones with prior antibiotic treatments^37^, across multiple antibiotic classes, is also consistent with that hypothesis.

Limitations of this study include the relatively small number of subjects to address associations with covariates. On average, the subjects in the USA were of higher socioeconomic status, more urban, and better-educated^38^ than the general population, so they were not fully representative of the USA population. Comparing fecal samples from the immediately post-partum women in the USA to populations of largely non-pregnant women is an intrinsic limitation as well. Individual dietary information was generally not available, so oxalate exposure differences could not be evaluated. Nevertheless, this study used the same *O*. *formigenes*-specific methodologies to examine specimens across three different populations in large cross-sectional surveys; this is the first report in the literature of such comparisons. In conclusion, *O*. *formigenes* was present at much lower prevalence in the USA subjects compared to remote indigenous groups, consistent with the hypothesis that *O*. *formigenes* is a part of the ancestral human gut microbiota, and that it may be disappearing in the context of socioeconomic advances and medical treatments, potentially contributing to the rise in nephrolithiasis.

## Methods

### Populations tested

All experiments were performed in accordance with relevant guidelines and regulations concerning human subjects (Table 2). United States. Fecal samples were collected from a cohort of mothers and infants in New York City as part of the Early Childhood Antibiotics and the Microbiome (ECAM) study^38^. The specimens used in this study were obtained under the ECAM protocol, approved by the NYU Langone Medical Center IRB, as reported in detail^38^. Informed consent was obtained from all subjects, or if subjects were under 18, from a parent and/or legal guardian. Forty of the 52 mothers enrolled in the study during their pregnancy self-collected fecal samples during the first month post-partum (Mean 9 +/−12 days (SD) post-partum). Fecal samples were collected from their infants within the first postpartum day and usually represented meconium, and then regularly through three years of life. Mothers were provided sterile supplies and instructions for sanitary collection of their own and their infant’s stools at home. Fecal samples were immediately chilled to −2 to −8 C, and delivered within 24–48 hours to the laboratory where they were frozen to −80 C. The maternal post-partum (n = 40) and infant delivery (n = 17) samples, as well as those obtained at ~6 months (n = 17), 12 m (n = 33), 18 m (n = 38), 24 m (n = 38), 30 m (n = 30) and 36 m (n = 26) were examined for *O*. *formigenes* colonization.

**Table 2:** Demographics of the 442 subjects in three locales.

Region	No. subjects	Age: range (y), mean ±SD	Sex: % female	BMI*: mean ±SD	Delivery: % vaginal	Race or Ethnicity: (%)	
Tanzania	182	0–80, 28.3 ± 19.7	48.3	119.5 ± 3.4	100	Hadza	100
Venezuela	178	0–62, 18.3 ± 16.2	54.5	18.9 ± 5.1	100	Sanema	54
						Yekwana	46
New York-Mothers	40	23–40, 35.8 ± 4.5	100	24.7 ± 4.3	NA	African-Am	5
						Asian	18
						Hispanic	10
						White	65
						Other	3
New York-Infants	42	0–3, NA^ŧ^	33.3	NA	55	African-Am	5
						Asian	17
						Hispanic	10
						White	67
						Other	2

*BMI included for subjects over age 5 years.

^ŧ^Samples tested at multiple time points over first three years of life.

### Tanzania

The Hadza live in north-central Tanzania around Lake Eyasi in the central Rift Valley. There are currently ~1,200 Hadza with fewer than 200 following a traditional foraging lifestyle that includes obtaining ~80–90% of their caloric needs from hunted and foraged resources. Fecal samples were collected from 182 traditional subjects of the Hadza tribe across wet and dry seasons^29^. The Hadza are among the last remaining hunter-gatherer populations in Africa, and reside in the eastern Rift Valley of Tanzania near Lake Eyasi^39^. Traditional Hadza live in camps averaging 20–30 people, with numbers varying according to season and availability of resources. Six camps were represented in this study (Kipamba, Makao, Mwamudu, Onowas, Sengeli, Ukamako)^29^. Until recently, Hadza had limited access to medical dispensaries and rural hospitals and thus western medications. However, recent encroachment into Hadzaland by pastoralists and other populations has increased access to western medical care – with medical posts within a day’s walk from the camps sampled. Permission for the study was obtained from the National Institute of Medical Research (MR/53i 100/83, NIMR/HQ/R.8a/Vol.IX/1542) and the Tanzania Commission for Science and Technology. As per our IRB, Informed Consent was obtained. Prior to sample collection, a brief explanation of the project and its objectives was given in a way that could be easily understood in Swahili via a translator. Each participant then signed a Consent Form either with a signature or thumb print in the presence of a witness to confirm her/his willingness to participate. In the case of minors, consent was provided by a parent or legal guardian^29^. The number of samples collected was not pre-specified for ensuring adequate statistical power. Subjects ranged from 6 months to 80 years of age; the age of six subjects was unknown and those samples were omitted from related analyses. Of the 182 subjects tested, 8 were pregnant women.

Fecal samples were self-collected by subjects, frozen in liquid nitrogen within 0–2 hours, and maintained frozen during all transport and storage until processed for analysis in the United States.

### Venezuela

Fecal samples were collected from 178 Amerindians in seven villages in the Yanomami-Sanema and Yekwana territory located in the rain forest of the High Caura river basin, from Bolivar state in Venezuela, under Venezuelan Institute of Scientific Research (IVIC) institutional review board (IRB) approval (project Dir0229/10 approval granted to M. Contreras)^40^. Informed consent was obtained according to the approved IRB protocol, first from the community leaders, then from the individual volunteers who were explained the study, and who signed or stamped their thumb print on the consent form. Parents signed to consent their infants and children. Villagers lack a market economy, and live in relative isolation in villages from 15 to 100 people, with access to the closest town by river requiring travel for one week. They maintain the traditional lifestyle of hunter-gatherer–gardeners common in the remote villages in this mountainous region. There are no health posts in Sanema villages, and one health post in one Yekwana village, while the others receive no medical care, except by infrequent visits from physicians. Amerindian subjects ranged from 3 months to 62 years of age; pregnant women were not sampled. Fecal samples were collected in the field and immediately frozen in liquid nitrogen, and remained frozen until the time of DNA extraction.

### PCR methods

DNA extraction from fecal swabs was performed using the PowerSoil DNA Extraction Kit (MoBio, Carlsbad CA), per the manufacturer’s protocol. All extracted DNA was stored at −80 C. Primers were designed based on the sequence alignment of the first 500 bp of the *oxc* gene of *O*. *formigenes*. The specificity of these primers [5′-ATGTAGAGTTGACTGATGGC-3′ (forward) and 5′-TTGATG CTGTTGATACG-3′ (reverse)] was confirmed by examining their homology with all other bacterial sequences deposited at the National Center for Biotechnology Information Basic Local Alignment Search Tool website (http://www.ncbi.nlm.nih.gov/BLAST). PCR was performed using Taq DNA Polymerase and 10x CoralLoad PCR buffer (Qiagen, Valencia CA). Each 50 ul reaction contained 1ul of extracted DNA, 0.2 uM of each primer and 6.3 uM DNTPs. PCR cycling conditions included an initial denaturation at 95 C for 3 min and 39 cycles of denaturation at 95 C for 30 s, annealing at 54 C for 30 s, and extension at 72 C for 1 min. PCR products were analyzed by electrophoresis using 2% agarose gels.

### Statistical Analysis

For the USA studies in which more extensive metadata were available, logistic regression was applied to explore the relationship between the *O*. *formigenes* status of each infant and of their mother. Analyses were performed with and without adjustment for the mother’s covariates (including age, BMI, race, antibiotic use prior to pregnancy, antibiotic use status during pregnancy, and antibiotics use status during delivery). Due to small sample size, race was defined as a binary variable with white = 1 and nonwhite = 0. Infants’ *O*. *formigenes* status was their status at each time point or being positive at any time. As a sensitivity analysis, the agreement of infant and mother *O*. *formigenes* status pair also was evaluated by Cohen’s kappa test statistic^41^. Fisher exact test, chi-squared test and logistic regression with or without adjustment for mother’s covariates were applied for testing whether the *O*. *formigenes* status of infants at each time or being positive at any time was related to their birth method, antibiotic use status, and gender, respectively. The generalized linear mixed model was used to determine whether the infants’ *O*. *formigenes* status during 0–36 months was related to their baseline variates and time dependent covariates such as antibiotic usage, with or without adjustment for their mother’s covariates. Time points were converted to 0–36 months, and missing data were omitted.

## Data Availability

The datasets generated during and/or analyzed during the current study are available from the corresponding author on reasonable request.

## References

[1] Allison MJ, Dawson KA, Mayberry WR, Foss JG (1985). Oxalobacter formigenes gen. nov., sp. nov.: oxalate-degrading anaerobes that inhabit the gastrointestinal tract. Archives of Microbiology.

[2] Allison MJ, Cook HM, Milne DB, Gallaher S, Clayman RV (1986). Oxalate degradation by gastrointestinal bacteria from humans. Journal of Nutrition.

[3] Cornick NA, Allison MJ (1996). Assimilation of oxalate, acetate, and CO2 by Oxalobacter formigenes. Can J Microbiol.

[4] Cornick NA, Allison MJ (1996). Anabolic Incorporation of Oxalate by Oxalobacter formigenes. Appl Environ Microbiol.

[5] Allison MJ, Dawson KA, Mayberry WR, Foss JG (1985). Oxalobacter formigenes gen. nov., sp. nov.: oxalate-degrading anaerobes that inhabit the gastrointestinal tract. Arch.Microbiol..

[6] Holmes RP (2000). Oxalate synthesis in humans: assumptions, problems, and unresolved issues. Mol.Urol..

[7] Holmes RP, Assimos DG (2004). The impact of dietary oxalate on kidney stone formation. Urol Res.

[8] Hoppe B (2011). Efficacy and safety of Oxalobacter formigenes to reduce urinary oxalate in primary hyperoxaluria. Nephrol Dial Transplant.

[9] Hoppe B, Dittlich K, Fehrenbach H, Plum G, Beck BB (2011). Reduction of plasma oxalate levels by oral application of Oxalobacter formigenes in 2 patients with infantile oxalosis. Am J Kidney Dis.

[10] Kaufman DW (2008). Oxalobacter formigenes may reduce the risk of calcium oxalate kidney stones. J Am Soc Nephrol.

[11] Hatch M, Gjymishka A, Salido EC, Allison MJ, Freel RW (2011). Enteric oxalate elimination is induced and oxalate is normalized in a mouse model of primary hyperoxaluria following intestinal colonization with Oxalobacter. American journal of physiology. Gastrointestinal and liver physiology.

[12] Hatch M, Freel RW (2013). A human strain of Oxalobacter (HC-1) promotes enteric oxalate secretion in the small intestine of mice and reduces urinary oxalate excretion. Urolithiasis.

[13] Hatch M (2006). Oxalobacter sp. reduces urinary oxalate excretion by promoting enteric oxalate secretion. Kidney Int.

[14] Arvans D (2017). Oxalobacter formigenes-Derived Bioactive Factors Stimulate Oxalate Transport by Intestinal Epithelial Cells. Journal of the American Society of Nephrology: JASN.

[15] Siener R (2013). The role of Oxalobacter formigenes colonization in calcium oxalate stone disease. Kidney Int.

[16] Hesse A, Siener R (1997). Current aspects of epidemiology and nutrition in urinary stone disease. World J Urol.

[17] Scales CD, Smith AC, Hanley JM, Saigal CS (2012). Prevalence of kidney stones in the United States. European urology.

[18] Dwyer ME (2012). Temporal trends in incidence of kidney stones among children: a 25-year population based study. The Journal of urology.

[19] Kittanamongkolchai W (2018). The Changing Incidence and Presentation of Urinary Stones Over 3 Decades. Mayo Clin Proc.

[20] Barnett C, Nazzal L, Goldfarb DS, Blaser MJ (2015). The Presence of Oxalobacter Formigenes in the Microbiome of Healthy Young Adults. The Journal of urology.

[21] Liu M (2017). Oxalobacter formigenes-associated host features and microbial community structures examined using the American Gut Project. Microbiome.

[22] Kwak C (2001). Molecular identification of Oxalobacter formigenes with the polymerase chain reaction in fresh or frozen fecal samples. BJU Int.

[23] Kodama T (2003). Detection of Oxalobacter formigenes in human feces and study of related genes in a new oxalate-degrading bacterium. Hinyokika Kiyo.

[24] Kumar R (2002). Role of Oxalobacter formigenes in calcium oxalate stone disease: a study from North India. European urology.

[25] Lange JN (2012). Sensitivity of human strains of Oxalobacter formigenes to commonly prescribed antibiotics. Urology.

[26] Kharlamb V (2011). Oral antibiotic treatment of Helicobacter pylori leads to persistently reduced intestinal colonization rates with Oxalobacter formigenes. J Endourol.

[27] Sidhu H (1998). Absence of Oxalobacter formigenes in cystic fibrosis patients: a risk factor for hyperoxaluria. Lancet.

[28] Kumar R, Ghoshal UC, Singh G, Mittal RD (2004). Infrequency of colonization with Oxalobacter formigenes in inflammatory bowel disease: possible role in renal stone formation. J Gastroenterol Hepatol.

[29] Smits SA (2017). Seasonal cycling in the gut microbiome of the Hadza hunter-gatherers of Tanzania. Science.

[30] Sidhu H (1997). Evaluating children in the ukrain for colonization with the intestinal bacterium Oxalobacter formigenes, Using a polymerase chain reaction based detection system. Molecular Diagnosis.

[31] Cornelius JG, Peck AB (2004). Colonization of the neonatal rat intestinal tract from environmental exposure to the anaerobic bacterium Oxalobacter formigenes. Journal of medical microbiology.

[32] Schnorr SL (2014). Gut microbiome of the Hadza hunter-gatherers. Nature communications.

[33] Turroni S (2016). Fecal metabolome of the Hadza hunter-gatherers: a host-microbiome integrative view. Sci Rep.

[34] Yatsunenko T (2012). Human gut microbiome viewed across age and geography. Nature.

[35] Clemente, J. C. *et al*. The microbiome of uncontacted Amerindians. *Science advances* **1**, 10.1126/sciadv.1500183 (2015). 10.1126/sciadv.1500183 PMC4517851 26229982

[36] Barnett C, Nazzal L, Goldfarb DS, Blaser MJ (2015). The presence of Oxalobacter formigenes in the microbiome of healthy young adults. The Journal of urology.

[37] Tasian, G. E., Jemielita, T., Goldfarb, D. S., Gerber, J. & Wu, Q. Oral antibiotic exposure and kidney stone disease. *J Amer Soc Nephrol* (2018). 10.1681/ASN.2017111213 PMC6054354 29748329

[38] Bokulich NA (2016). Antibiotics, birth mode, and diet shape microbiome maturation during early life. Science translational medicine.

[39] Marlowe, F. *The Hadza: Hunter-Gatherers of Tanzania*. First edition edn, (University of California Press, 2010).

[40] Ruggles K (2018). Changes in the gut microbiota of urban subjects during an immersion in the traditional diet and lifestyle of a rainforest village. mSphere.

[41] Landis JR, Koch GG (1977). The measurement of observer agreement for categorical data. Biometrics.

